# Crimean-Congo Hemorrhagic Fever Virus IgG in Goats, Bhutan

**DOI:** 10.3201/eid2205.151777

**Published:** 2016-05

**Authors:** Sonam Wangchuk, Sonam Pelden, Tenzin Dorji, Sangay Tenzin, Binay Thapa, Sangay Zangmo, Ratna Gurung, Kinzang Dukpa, Tenzin Tenzin

**Affiliations:** Ministry of Health Public Health Laboratory, Thimphu, Bhutan (S. Wangchuk, S. Pelden, T. Dorji, B. Thapa, S. Zangmo);; Ministry of Agriculture and Forests National Centre for Animal Health, Thimphu (S. Tenzin, R. Gurung, K. Dukpa, T. Tenzin)

**Keywords:** Crimean-Congo hemorrhagic fever, viral hemorrhagic fever, tick-borne disease, nairovirus, viruses, IgG, antibodies, domestic animals, ruminants, goats, serosurvey, Bhutan, vector-borne infections

**To The Editor:** Crimean-Congo hemorrhagic fever (CCHF) is a highly infectious tickborne disease caused by a high-risk group of viruses belonging to the family *Bunyaviridae* (*1**,**2*). In humans, the overall case-fatality rate of CCHF is ≈30%, but in severe and hospitalized patients, fatalities may be up to 80% (*3**,**4*). CCHF is widespread in various countries in Africa, Asia, and Europe; the virus had been identified in humans in China, Pakistan, and Afghanistan and has been recently reported for the first time in humans in India (*4**–**7*). Humans can be infected by bites from infected ticks, mainly of the *Hyalomma* genus; by unprotected contact with blood or tissue of viremic patients; or during slaughtering of infected animals. In addition, nosocomial infections are found in humans (*1**,**4**,**8**,**9*).

Fatal cases of CCHF in humans were confirmed in Ahmadabad in India in 2011, but a recent serosurvey in livestock showed that this disease has widespread seroprevalence in domestic animals across India (*7**–**10*). Bhutan shares a long, porous border with India, and animals and humans frequently cross the border. Comprehensive surveillance was needed to determine the presence of CCHF virus (CCHFV) in livestock in Bhutan and to assess risk for zoonotic infection in humans.

During October 2015, in collaboration with the National Centre for Animal Health Bhutan, we retrospectively tested serum samples collected during April–May 2015 from 81 goats and 92 cattle for CCHFV-specific IgG by using ELISA kits (Sheep/goat anti-CCHFV IgG ELISA kit and Cattle anti-CCHFV IgG ELISA kit; National Institute of Virology, Pune, India), as described (*10*). CCHFV IgG was detected in 31 (38.2%) goats; no cattle had positive results. The samples from goats, which were collected in early 2015 as part of surveillance of peste des petits ruminants, originated from the southern district of Sarpang, which shares a porous border with the state of Assam in India (Figure). The samples from cattle were collected from the National Nublang Breeding Center (Trashigang district) and the National Jersey Breeding Center (Samtse district) (Figure). Findings indicated that all goats that tested positive for CCHFV were reported to have been either bred within households that kept goat herds or procured from other villages within the district. Exact sources of those seropositive goats could not be ascertained. However, in a few instances in the past, breeding goats (male and female) were procured from India by the Bhutan government and distributed to farmers for breed improvement. We also believe that cross-border movement of animals and unofficial imports of goats by farmers along the porous borders of southern Bhutan likely occurred. Furthermore, a large number of dairy cattle are imported annually from India for enhancing milk production and breeding purposes. Not all imported animals (both cattle and goats) were tested for CCHF because of a lack of diagnostic facilities and the negligible occurrence of the disease in livestock. 

**Figure:**
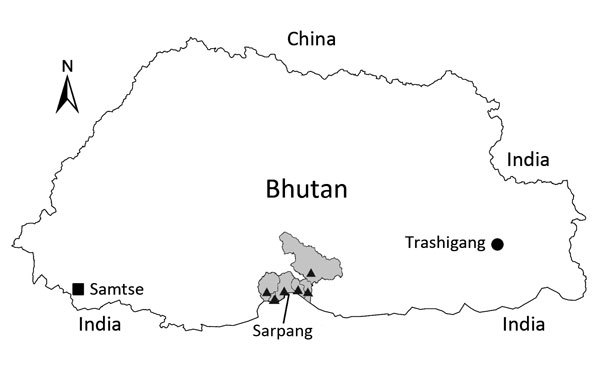
Locations in Bhutan where serum samples were collected from goats (triangles) and cattle (square and circle) and tested for Crimean-Congo hemorrhagic fever virus. The shaded area shows the boundaries of Sarpang district and subdistricts, where samples from goats were collected.

Our findings indicate that the risk of importing emerging infectious diseases along with live animals poses a serious risk to public health. Consequently, detailed risk-based surveillance is necessary to understand the complete scenario of CCHFV prevalence in livestock in Bhutan because *Hyalomma* tick species, the primary vectors of CCHF, are present on animals here. In addition, a survey among at-risk human populations is also needed. Findings from these surveillance activities would help institute more diagnostic facilities and risk-based surveillance and assist in developing a preparedness plan at the human–animal interface. Although our study has limitations because of the low number of serum samples tested from limited animal species from only 3 areas, the study provides evidence that CCHFV is circulating in goats in Bhutan.
